# Cargo Stability of Extracellular Vesicles Derived From Non‐Diseased Sources: Effects of Storage and Handling Conditions

**DOI:** 10.1111/jcmm.71411

**Published:** 2026-10-29

**Authors:** Khadijeh Abbasi, Mina Mohammadinasr, Roza Motavalli, Narges Arianfar, Attabak Toofani‐Milani, Moein Shirzad, Vahid Hosseini, Masoud Darabi

**Affiliations:** ^1^ Department of Clinical Biochemistry and Clinical Laboratory, Faculty of Medicine Tabriz University of Medical Sciences Tabriz Iran; ^2^ Molecular Medicine Research Center Tabriz University of Medical Sciences Tabriz Iran; ^3^ Department of Pharmaceutical Biotechnology, Faculty of Pharmacy Tabriz University of Medical Sciences Tabriz Iran; ^4^ Department of Medical Laboratory Sciences and Microbiology, TaMS.C Islamic Azad University Tabriz Iran; ^5^ Cellular and Molecular Biology Research Center, Health Research Institute Babol University of Medical Sciences Babol Iran; ^6^ University of Luebeck | Department of Hematology and Oncology University Cancer Center Schleswig‐Holstein (UCCSH) and University Hospital Schleswig‐Holstein (UKSH) Luebeck Germany

**Keywords:** biofluids, biological specimen bank, cell communication, cell‐derived microparticles, exosomes, specimen handling

## Abstract

Extracellular vesicles (EVs) are nanoscale, membrane‐enclosed particles that transfer proteins, nucleic acids, lipids, and other bioactive molecules between cells. Their cargo‐bearing capacity supports applications in drug delivery, regenerative medicine, and biomarker development, but ex vivo handling can alter vesicle recovery and cargo readouts. Storage temperature, pH, freeze–thaw exposure, storage duration, and buffer composition differentially affect EV‐associated proteins and nucleic acids. This review evaluates preservation studies using EVs from non‐diseased cells, tissues, and biological fluids, with emphasis on the distinction between EV recovery, cargo abundance, molecular integrity, and functional preservation. Across the available literature, optimal conditions depend on EV source, biological matrix, isolation method, storage duration, cargo class, and analytical endpoint. Standardized protocols that separately measure vesicle recovery, cargo abundance, molecular integrity, and biological activity are required to define source‐ and application‐specific preservation strategies and resolve the mechanisms of storage‐associated cargo alteration.

## 1 Introduction

Extracellular vesicles (EVs) mediate intercellular communication by transferring molecular cargo between donor and recipient cells [1, 2]. These nanoscale, membrane‐enclosed particles participate in tissue repair, immune regulation, and disease‐associated signalling, including EV‐mediated communication in cancer models [3, 4, 5, 6]. EVs are released by many cell types and carry biologically active ribonucleic acid (RNA), deoxyribonucleic acid (DNA), proteins, lipids, and metabolites [2].

EV‐based diagnostic and therapeutic applications require preservation of vesicle integrity and cargo during ex vivo handling and storage [7, 8]. Temperature, pH, storage duration, buffer composition and repeated freeze–thaw exposure can alter EV recovery, membrane properties and the abundance or integrity of EV‐associated biomolecules. Preservation protocols therefore need to maintain reproducible vesicle recovery and biological activity across experimental, pharmaceutical and clinical workflows [9, 10].

EVs from non‐diseased cells and tissues are relevant to therapeutic development because their molecular composition reflects donor‐cell physiology and may carry fewer disease‐associated liabilities than EVs derived from pathological sources [11]. EV cargo nevertheless does not passively replicate the parental cell. Selective sorting mechanisms enrich or exclude specific proteins, nucleic acids and lipids during EV biogenesis, producing vesicle cargo profiles that can differ substantially from the source cell.

The EV phospholipid bilayer shields intraluminal cargo from extracellular enzymes and physicochemical stressors [12]. This barrier is particularly relevant for proteins and nucleic acids, which are vulnerable to enzymatic degradation, temperature‐dependent damage, pH‐dependent structural changes and prolonged storage. Preservation of vesicle number or morphology alone cannot establish preservation of cargo integrity or biological activity. EV recovery, cargo abundance, cargo integrity, and functional preservation must therefore be evaluated as separate endpoints when storage conditions are compared.

Standardized pre‐analytical and biobanking procedures are required to improve reproducibility and support clinical translation of EV‐based assays and products [13, 14]. Most EV studies focus on pathological conditions, including cancer, inflammation and degenerative disorders, whereas EVs from non‐diseased sources have received less focused analysis [15, 16, 17]. Defining the preservation requirements of these sources is essential for reproducible studies of physiological vesicle communication and for the development of safer EV‐based therapeutic platforms [18, 19].

This review critically evaluates studies of EVs derived from non‐diseased human and animal cells, tissues, and biological fluids. We first examine EV cargo composition and compositional differences between EVs and their parental cells, then assess how post‐isolation preservation conditions affect protein and nucleic acid cargo. The analysis distinguishes EV recovery, cargo abundance, molecular integrity and functional preservation, with emphasis on methodological and biological variables that explain divergent findings across studies.

## 2 EV Cargo Composition and Intercellular Transport

Extracellular vesicles carry proteins, lipids, nucleic acids, ions, and small signalling molecules. Donor‐cell physiology shapes this molecular composition and contributes to EV functions in intercellular communication, immune regulation, and metabolic and neurological signalling [20]. EVs released under physiological conditions commonly contain proteins involved in vesicle biogenesis, membrane organization, and cargo trafficking, including tetraspanins, endosomal sorting complex required for transport (ESCRT) components, ALG‐2‐interacting protein X (Alix), milk fat globule‐EGF factor 8, caveolin‐1 and flotillin‐2 [21]. EV preparations also contain immune‐related and trafficking‐associated proteins, including major histocompatibility complex (MHC) class I molecules, immunoglobulins, Rab GTPases, annexins and nucleic acid‐binding proteins [21, 22]. This molecular diversity reflects the heterogeneous functions of EVs and their capacity to mediate selective communication between donor and recipient cells.

### 2.1 Protein Cargo of EVs Derived From Non‐Diseased Cells

EV subpopulations, including small extracellular vesicles (sEVs) and large extracellular vesicles (lEVs), can differ substantially in their protein composition. This heterogeneity reflects differences in EV biogenesis, membrane organization, and cargo sorting and may contribute to distinct biological functions. Membrane raft‐associated proteins, including caveolin‐1, β‐actin, α‐actinin‐4 and flotillin‐2, have been reported to be enriched in lEVs, whereas sEVs are more frequently associated with endosomal sorting complex required for transport (ESCRT)‐related proteins, Alix and tetraspanins [21, 23, 24].

Comparative proteomic analyses of adipocyte‐derived EVs further illustrate subtype‐specific protein distribution. Fatty acid‐binding protein 4/adipocyte protein 2 (FABP4/aP2), annexin A2 and endoplasmin have been associated predominantly with lEVs, whereas adiponectin, fatty acid synthase and major vault protein (MVP) have been identified more frequently in sEV fractions [21, 25, 26, 27, 28, 29]. These compositional differences suggest that EV subpopulations may participate in distinct aspects of metabolic regulation, including lipid and glucose homeostasis. However, the functional consequences of subtype‐specific protein enrichment remain incompletely understood and require direct functional validation.

Protein heterogeneity is also evident in EVs derived from immune cells. EVs released by monocyte‐derived dendritic cells (DCs) contain major histocompatibility complex (MHC) class I and II molecules in both small and large EV populations. However, glycoprotein 96 (GP96) has been reported to be enriched in lEVs, whereas syntenin‐1, tumour susceptibility gene 101 (TSG101), a disintegrin and metalloprotease domain‐containing protein 10 (ADAM10), and EH domain‐containing protein 4 (EHD4) are more frequently associated with sEVs [21, 25, 26]. These differences support the existence of molecularly distinct EV subpopulations and may contribute to differences in antigen presentation and immune signalling.

EVs derived from DCs and macrophages can also carry cytokines and other immunoregulatory proteins involved in T‐cell activation and macrophage responses. Cytokines associated with EV fractions include interferon‐α (IFN‐α), interferon‐γ (IFN‐γ), tumour necrosis factor‐α (TNF‐α), and several interleukins (ILs) [30]. EV‐associated cytokine packaging may protect these molecules from extracellular degradation and facilitate localized or longer‐range signalling. Cytokines such as IL‐2, IL‐4, IL‐12, IFN‐γ, transforming growth factor‐β (TGF‐β), and TNF‐α have been detected in association with EVs, although their distribution between vesicular and non‐vesicular fractions varies among tissues and biological fluids, as summarized in Table 1 [31].

**TABLE 1:** Representative protein cargo identified in EVs derived from different cellular and biological sources.

EV source	Major EV‐associated proteins/cytokines	References
3T3‐L1 adipocytes/adipocyte‐derived EVs	Tetraspanins (CD9, CD63, CD81), trafficking proteins (Alix, Rab proteins, flotillin‐2), cytoskeletal proteins (Actin, tubulin, α‐actinin, myosin), histones, immune‐related proteins (MHC I, immunoglobulins) and metabolic proteins (FABP4, adiponectin, fatty acid synthase, syntenin‐1)	[21, 22, 23, 24, 25, 26, 27, 28, 29]
Placental villous tissue	IL‐33	[31]
T cells	Inflammatory cytokines, including IL‐2, IL‐4, IL‐12p70, IL‐17, IL‐21, IL‐22, IFN‐γ, TGF‐β, TNF‐α and ITAC	[31]
Monocytes	Inflammatory cytokines and chemokines, including IL‐1, IL‐2, IL‐4, IL‐12p70, IL‐17, IL‐21, IL‐22, IL‐33, IFN‐γ, TGF‐β, TNF‐α, ITAC and GRO‐α	[31]
Amnion	Pro‐inflammatory cytokines and immune mediators, including IL‐1α, IL‐15, IL‐16, IL‐18, GM‐CSF, M‐CSF, MIG and calgranulin A	[31]
Plasma	Immune‐related proteins, including IL‐10, RANTES and ITAC	[31]
Amniotic fluid	Cytokines and chemokines, including IL‐4, IL‐6, IL‐10, IL‐12, IL‐17 and MIP‐1α	[31]
Cervical explants	IL‐12, IL‐6 and IL‐4	[31]
Monocyte‐derived dendritic cells	Canonical EV markers (CD63, CD9, CD81, TSG101), MHC molecules and membrane trafficking/cytoskeletal proteins, including ADAM10, EHD4, syntenin‐1, actinin‐4 and mitofilin	[25]
Macrophages	IFN‐α, IFN‐γ, TNF‐α and interleukins	[30]
Reticulocytes	Membrane proteins, including transferrin receptor, CD55, CD58, and CD59 and lipid raft‐associated proteins, including stomatin and flotillin‐1/2	[32]
mpkCCD cells, apical EVs	Cytoskeletal proteins (α‐actinin‐1, moesin, filamin‐A), membrane trafficking proteins (annexins, clathrin heavy chain 1), chaperone proteins (HSP90), metabolic enzymes (GAPDH, α‐enolase) and signalling proteins (14–3‐3 ζ/δ)	[33]
LLC‐PK1 proximal tubule cells	GAPDH	[34]
Prostate gland epithelial cells	Prostate‐associated membrane and secretory proteins, including CD13, CD26, prostate‐specific antigen, prostatic acid phosphatase and zinc α2‐glycoprotein	[35]
Urine	Renal transport proteins, including prostasin, NKCC2, NCC, ENaC, AQP1, AQP2, pendrin, V‐ATPase and NHE3	[36, 37, 38]

Abbreviations: ADAM10, a disintegrin and metalloproteinase domain‐containing protein 10; Alix, ALG‐2‐interacting protein X; AQP, aquaporin; ENaC, epithelial sodium channel; EVs, extracellular vesicles; FABP4, fatty acid‐binding protein 4; GAPDH, glyceraldehyde‐3‐phosphate dehydrogenase; GM‐CSF, granulocyte–macrophage colony‐stimulating factor; GRO‐α, growth‐regulated oncogene‐α; IFN, interferon; IL, interleukin; ITAC, interferon‐inducible T‐cell α chemoattractant; M‐CSF, macrophage colony‐stimulating factor; MHC, major histocompatibility complex; MIG, monokine induced by IFN‐γ; NCC, sodium chloride cotransporter; NHE3, sodium–hydrogen exchanger 3; NKCC2, sodium–potassium–chloride cotransporter 2; PD‐L1, programmed death‐ligand 1; TGF‐β, transforming growth factor‐β; TNF‐α, tumour necrosis factor‐α; TSG101, tumour susceptibility gene 101.

Selective protein cargo distribution has also been observed in EVs derived from other non‐diseased cell types. During erythroid maturation, reticulocyte‐derived exosomes contain proteins such as transferrin receptor (CD71), flotillin‐1, stomatin and CD58, which are associated with membrane remodelling and differentiation [32]. In polarized epithelial cells, EV secretion and cargo composition can vary according to the membrane domain of origin. For example, exosomes released from the apical surface of mouse cortical collecting duct cells are enriched in α‐actinin‐1, moesin, annexins A1–A6 and glyceraldehyde‐3‐phosphate dehydrogenase (GAPDH), whereas basolaterally released EVs show distinct proteomic profiles. This polarity‐dependent cargo distribution may contribute to the differential regulation of recipient‐cell pathways, including epithelial sodium channel activity and renal fluid balance [33, 34].

Temporal variation in EV‐associated proteins has also been reported. Levels of the sodium chloride cotransporter (NCC) and prostasin in urinary EVs exhibit circadian fluctuations, suggesting that sampling time may influence the interpretation of urinary EV protein profiles. Such temporal variation may also be relevant to the evaluation of treatment responses, including those involving thiazide‐sensitive pathways in hypertension [37].

Together, these studies show that EV protein composition varies with vesicle subtype, donor‐cell identity, membrane polarity, and physiological context. Proteomic and comparative analyses support selective enrichment of specific proteins in EVs rather than passive release of cellular by‐products. Protein abundance alone, however, should not be treated as evidence of distinct biological function without direct functional validation.

### 2.2 Ionic Cargo of EVs

The ionic composition of EVs remains less characterized than their protein, lipid, and nucleic acid cargo. EVs may reflect donor‐cell ion homeostasis, but selective ion loading and the functional consequences of EV‐associated ions remain poorly defined. Although evidence under strictly physiological conditions is scarce, altered ion‐homeostasis models show that EVs can transport inorganic cargo. Furmanik et al. [39] reported that activation of calcium‐dependent NADPH oxidase 5 (Nox5) in human vascular smooth muscle cells (VSMCs) increased reactive oxygen species (ROS) production and promoted a contractile‐to‐synthetic phenotypic transition.

This transition was accompanied by increased release of calcium‐enriched EVs that transferred mineral‐associated cargo and promoted extracellular calcification [39]. Increased intracellular calcium and ROS signalling may reinforce EV release, generating a feed‐forward mechanism that links altered ion homeostasis, vesicle secretion, and mineral deposition. These data indicate that EVs can transport inorganic cargo and that donor‐cell ion metabolism can modify EV composition and downstream biological effects.

Evidence defining the ionic composition of EVs from non‐diseased sources remains limited. Quantitative studies are needed to determine whether ions are selectively incorporated into EVs, how ionic cargo differs from parental‐cell ion pools, and whether EV‐associated ions contribute to physiological intercellular communication. Better‐defined ionic cargo profiles may also clarify how ions regulate EV membrane properties and vesicle function.

### 2.3 Lipid Composition of EVs Derived From Non‐Diseased Cells

Lipids are integral to the structural organization and biological functions of extracellular vesicles. In addition to maintaining membrane integrity, curvature and fluidity, EV lipids can influence vesicle formation, cellular uptake and signalling in recipient cells. Unlike many other EV cargo components, lipids may undergo continuous enzymatic and non‐enzymatic remodelling. Donor‐cell lipid composition varies with metabolic and differentiation state [40, 41], which may in turn influence EV lipid composition [40]. Many EV preparations contain substantial amounts of saturated fatty acids, whereas specific unsaturated fatty acids, including docosahexaenoic acid (DHA), may be present at lower abundance but contribute to membrane organization and cholesterol metabolism [42, 43, 44].

EV subpopulations can exhibit distinct lipid profiles that reflect differences in their biogenesis and membrane origin. Exosomes and microvesicles (MVs) derived from human bone marrow mesenchymal stromal cells (MSCs), for example, differ in their lipid composition. Exosomes have been reported to be enriched in glycolipids, phosphatidylserine (PS), and free fatty acids, whereas these lipid classes are less abundant or unchanged in MVs. EV fractions may also contain relatively high proportions of long‐chain and polyunsaturated lipids [40, 45]. In contrast, cardiolipins, ceramides, lysophosphatidylethanolamines (LPEs), acylcarnitines and lysophosphatidylcholines (LPCs) have been reported at higher abundance in MVs than in exosomes [45]. These differences likely reflect distinct membrane origins, biogenesis pathways, and lipid‐sorting processes and may also be influenced by the metabolic state of the donor cells.

In addition to their structural roles, EV‐associated lipids can function as bioactive mediators. Anandamide, also known as N‐arachidonoylethanolamine (AEA), has been identified in microglia‐derived EVs and can activate cannabinoid receptor 1, thereby modulating presynaptic neuronal activity [46]. Comparative analyses of exosomes derived from reticulocytes, B lymphocytes, mast cells and dendritic cells have also demonstrated enrichment of cholesterol (Chol), sphingomyelin (SM), PS, LPEs, LPCs, phosphoinositides (PIs) and the ganglioside GM3 [47, 48, 49, 50]. Such compositional patterns support the involvement of EV lipids in membrane organization and intercellular signalling.

EVs can also transport immunomodulatory lipid mediators. Prostaglandin E2 (PGE2) associated with intestinal EVs has been implicated in regulation of the gut–liver immune axis and induction of natural killer (NK) cell anergy through Wingless/Integrated (Wnt)‐related signalling pathways [51, 52]. Similarly, EV‐associated sphingosine‐1‐phosphate (S1P) produced in response to enterobacterial stimulation can contribute to T helper 17 (Th17) cell activation [53, 54]. Leukotrienes, including leukotriene B4 (LTB4) and leukotriene C4 (LTC4), have been identified in EVs derived from macrophages, plasma, and dendritic cells, and can promote granulocyte chemotaxis [55].

Lipid remodelling in donor cells may further influence the biological activity of released EVs, particularly in metabolically active secretory tissues such as adipose tissue [56]. Alterations in polyunsaturated fatty acid (PUFA) metabolism in human mesenchymal stromal cells have been associated with increased production of anti‐inflammatory lipid mediators, including resolvins and PGE2, some of which are transported in EVs and may contribute to their immunomodulatory effects [57]. EVs derived from mast cells can similarly contain arachidonic acid‐derived mediators, including PGE2 and 15‐deoxy‐Δ12,14‐prostaglandin J2 (15d‐PGJ2) [57, 58]. In the nervous system, sphingomyelin metabolism within MVs can generate ceramide and sphingosine, which have been linked to modulation of glutamatergic signalling [42, 43]. These observations illustrate that EV‐associated lipids can act not only as membrane constituents but also as active regulators of recipient‐cell responses.

Source‐specific and membrane‐domain‐specific lipid sorting has also been reported. Platelet‐derived EV subfractions exhibit distinct lipid profiles, including differences in sphingomyelin, ceramide, phosphatidylserine, and lysophosphatidylcholine abundance [53]. In polarized epithelial cells, basolaterally released exosomes are relatively enriched in SM, phosphatidylcholine (PC), phosphatidylglycerol (PG) and PS, whereas apically released exosomes show higher cardiolipin abundance. Ceramide and monoalkyldiacylglycerol levels also differ between apical and basolateral EV populations, supporting membrane‐domain‐specific lipid sorting [33].

EV lipid composition can also differ substantially from that of the surrounding biological fluid. Human plasma‐derived exosomes contain monoacylglycerols (MAGs), triacylglycerols (TAGs), diacylglycerols (DAGs), ceramides, PCs, LPCs and PIs. TAG and DAG have been reported to be enriched in exosome fractions relative to plasma, suggesting that EVs may contribute to lipid transport through mechanisms distinct from conventional lipoprotein‐mediated pathways [59]. Human milk‐derived EVs also display source‐ and developmental‐stage‐dependent lipid profiles. EVs from preterm milk contain higher levels of SM, TAG and selected PC subclasses, whereas term milk EVs show enrichment of other PC and phosphatidylethanolamine (PE) subclasses [60]. These compositional differences may reflect adaptations to the developmental and nutritional requirements of preterm and full‐term infants.

Adipocyte‐derived EVs provide a further example of functional lipid transfer, consistent with the broader secretory and endocrine functions of white, beige and brown adipose tissues [56]. These EVs can transport TAG to bone marrow‐derived cells and influence their differentiation toward adipose tissue macrophage‐like phenotypes, supporting a role for EV‐mediated lipid transfer in metabolic homeostasis and immune regulation [61].

EV lipids have structural and signalling functions. Their composition varies with EV subtype, membrane origin, donor‐cell metabolism and physiological context. Cholesterol, sphingolipids, phospholipids and bioactive lipid mediators shape EV membrane properties and may modulate recipient‐cell responses after vesicle uptake. Lipid abundance alone, however, should not be interpreted as evidence of a specific biological function without direct functional validation. More rigorous lipidomic characterization of EVs from non‐diseased sources will support biomarker discovery and the design of EV‐based delivery systems (Table 2).

**TABLE 2:** Representative lipid cargo identified in EVs derived from different cellular and biological sources.

EV source	Major EV lipid cargo	Reported or proposed biological function	References
Intestinal mucosa	PGE2	Induction of hepatic natural killer (NK) cell anergy	[51, 52]
Macrophages and dendritic cells	LTC4, LTB4	Promotion of granulocyte migration	[55]
Plasma	LTB4	Promotion of granulocyte migration	[55]
Intestinal epithelium	S1P	Regulation of T helper 17 (Th17) cell activity	[54]
Microglial cells	Anandamide	Activation of cannabinoid receptor 1 (CB1) and inhibition of presynaptic transmission	[46]
B lymphocytes	Chol, SM, PS, PC, PE, PA, PI	Structural components of EV membranes; functional role not directly assessed	[42]
Mast cells	Chol, SM, PS, PC, PE, PI	Structural components of EV membranes; functional role not directly assessed	[42]
Dendritic cells	Chol, SM, PS, PC, PE	Structural components of EV membranes; functional role not directly assessed	[42]
Reticulocytes	Chol, SM, PS, PC, PE, PI	Structural components of EV membranes during reticulocyte maturation	[42]
Platelets	Chol, SM, PS, PC, PE, PI, Cer	Structural and bioactive lipid components of platelet‐derived EVs	[42, 53]
Adipocytes	Chol, SM, PS, PC, PE, PI, Cer, HexCer, DAG	Lipid transport and metabolic communication	[42]
Oli‐neu cells	Chol, SM, PS, PC, PE	Structural components of EV membranes; functional role not directly assessed	[42]
Human plasma	TAGs, DAGs, MAGs, Cer, PC, SM, LPC, PI, sulfatides	Potential role in extracellular lipid transport and intercellular lipid signalling	[59]
Human bone marrow‐derived mesenchymal stromal cells	Chol, SM, PS, PC, PI	EV subtype‐specific membrane organization and lipid composition	[45]

Abbreviations: Cer, ceramide; Chol, cholesterol; DAG, diacylglycerol; HexCer, hexosylceramide; LPA, lysophosphatidic acid; LPC, lysophosphatidylcholine; LTB4, leukotriene B4; LTC4, leukotriene C4; MAG, monoacylglycerol; PA, phosphatidic acid; PC, phosphatidylcholine; PE, phosphatidylethanolamine; PGE2, prostaglandin E2; PI, phosphatidylinositol; PS, phosphatidylserine; S1P, sphingosine‐1‐phosphate; SM, sphingomyelin; TAG, triacylglycerol.

## 3 Selective Cargo Sorting and Compositional Differences Between EVs and Their Biological Sources

Although EVs and their parent cells share biomolecules such as cytoplasmic proteins and lipids, their molecular composition can differ substantially. Specific proteins, nucleic acids and lipids may be selectively enriched or excluded during EV biogenesis, yielding cargo profiles that do not simply mirror the parental cell. These compositional differences are consistent with regulated cargo‐sorting mechanisms and may influence the biological effects of EVs on recipient cells.

Analysis of amino acids in EV‐enriched fractions isolated from human serum provides one example of differential cargo distribution. Although the major amino acids detected in EV fractions were also present in native serum, their relative abundances differed between the two compartments [62]. Several amino acids, including His, Arg, Glu, Cys, Lys and Tyr, were relatively enriched in EV fractions, whereas Asn, Gln and Ala showed lower relative abundance. D‐Ser was also enriched in EV fractions compared with native serum [62]. These findings suggest that the small‐molecule composition of EVs may differ from the surrounding biological fluid. Such differences may reflect the metabolic state of donor cells, EV biogenesis pathways or associations with selectively incorporated protein cargo rather than passive incorporation alone.

Changes in EV composition during cellular maturation provide further evidence of regulated cargo sorting. During reticulocyte maturation, transferrin receptors are removed from the plasma membrane through their incorporation into released vesicles, contributing to the transition toward mature erythrocytes [63]. Despite this selective removal of membrane proteins, the overall phospholipid composition and aminophospholipid distribution of reticulocyte‐derived EVs remain partly similar to those of their parental cells [47]. Thus, EV formation may combine selective cargo removal with preservation of broader membrane characteristics.

A similar pattern has been observed during adipocyte differentiation. EVs released from differentiating 3T3‐L1 cells showed changes in phosphatidylserine exposure, total fatty acid content and phospholipid composition that differed from the corresponding changes in parental cells [64]. Whereas fatty acid abundance increased substantially in both cells and EVs during differentiation, the magnitude and species‐level pattern of these changes differed between the two compartments. Phosphatidylcholine, phosphatidylinositol, sphingomyelin and phosphatidylethanolamine also showed differential remodelling in cells and EVs [64]. These observations indicate that metabolic remodelling of donor cells influences EV lipid cargo but does not necessarily produce a proportional copy of the parental‐cell lipidome.

The biological matrix from which EVs are isolated can also substantially influence the apparent cargo profile. Comparisons of serum‐ and plasma‐derived EV fractions have identified differences in particle abundance and proteomic composition, with serum EV fractions showing greater representation of platelet‐associated proteins in some studies [65]. This finding is consistent with platelet activation and vesicle release during blood coagulation. However, another proteomic study reported higher abundance of EV‐associated marker proteins in plasma‐derived EV preparations [66]. These apparently conflicting observations may reflect differences in sample processing, EV isolation, mass spectrometry platforms and data‐analysis workflows. They also emphasize that serum and plasma EV preparations should not be considered directly interchangeable when comparing EV cargo profiles.

Polarized epithelial cells provide a further example of spatially regulated EV cargo sorting. Madin‐Darby canine kidney (MDCK) cells release distinct EV populations from their apical and basolateral surfaces [67]. Wnt3a released from the basolateral side was associated with TSG101‐containing EV fractions, whereas apically released Wnt3a and Wnt11 were predominantly detected in CD63‐ and Hsp70‐associated EV fractions of different densities [67]. These findings suggest that cell polarity and asymmetric membrane trafficking influence the incorporation of signalling molecules into specific EV populations. Thus, the intracellular route of cargo transport and the site of EV release may contribute to EV heterogeneity independently of the overall composition of the donor cell.

Selective RNA loading has also been demonstrated in mast cell‐derived EVs. EVs released by MC/9, HMC‐1, and bone marrow‐derived mast cells exhibited RNA profiles that differed from those of their parental cells, including relatively low ribosomal RNA content and distinct messenger RNA (mRNA) and microRNA (miRNA) distributions [68]. A subset of mRNA transcripts and miRNAs was selectively enriched or detected predominantly in EVs. These observations indicate that EV‐associated RNA does not represent a random sample of cellular RNA and support the existence of selective RNA‐sorting mechanisms during EV formation.

Differences between EV and parental‐cell proteomes have similarly been observed in HEK293T cells. EV preparations were enriched in EV‐associated proteins such as CD63, CD9 and TSG101 and showed a greater representation of cytoplasmic and extracellular proteins, whereas parental‐cell lysates contained a broader range of nuclear and mitochondrial proteins [69]. Proteins associated with RNA processing and protein synthesis also showed differential enrichment between EVs and cells. These proteomic differences further support selective incorporation or exclusion of proteins during EV biogenesis rather than passive sampling of the cellular proteome.

Compositional heterogeneity can occur even among EVs released by a single donor‐cell type. Dendritic cell‐derived EVs contain shared proteins, including major histocompatibility complex proteins and Hsp70, but EV populations enriched in CD63, CD81, or CD9 exhibit distinct protein profiles [25]. Further fractionation and proteomic analyses identified multiple EV subpopulations with different molecular compositions. These observations challenge the assumption that EVs released from one cell type form a homogeneous population and highlight the need for EV subtype‐specific characterization and standardized nomenclature.

Comparative lipid analyses provide additional evidence of selective membrane organization. EVs derived from rat mast cells and human dendritic cells showed differences in sphingomyelin and phosphatidylcholine abundance compared with their parental cells, whereas cholesterol levels were relatively similar [49]. EV membrane rigidity was also influenced by environmental pH, and lipid movement between membrane leaflets differed from that of the parental‐cell plasma membrane. Nevertheless, EVs retained the major phospholipid classes present in cellular membranes [49]. Together, these findings indicate that EV membranes retain fundamental characteristics of parental‐cell membranes while undergoing selective lipid remodelling that may influence membrane organization and function.

However, EV‐related particles do not invariably exhibit marked compositional divergence from their cells of origin. Pre‐osteoclast and mature osteoclast apoptotic bodies showed RNA profiles largely resembling those of the corresponding parental cells, with mRNA and long non‐coding RNA forming the predominant RNA cargo [70]. Their functional effects also reflected the differentiation state of the originating cells: pre‐osteoclast apoptotic bodies promoted angiogenic responses, whereas mature osteoclast apoptotic bodies enhanced osteogenic differentiation. Subtle differences in RNA profiles nevertheless remained dependent on the developmental state of the parental cells [70]. These findings indicate that the extent of cargo selectivity may vary according to vesicle type and biogenesis pathway.

Similarly, phospholipid analysis of packed red blood cells (RBCs) and their released EVs during blood‐bank storage revealed substantial similarities in membrane lipid composition [71]. No marked enrichment of phosphatidylserine or sphingomyelin was observed in EVs, and the cholesterol‐to‐phospholipid relationship remained comparable between EVs and RBCs. Nevertheless, differences in fatty acyl chain composition suggested a degree of selective lipid incorporation during EV release [71]. Thus, even when the global molecular composition of EVs resembles that of the parental cell, subtler species‐level differences may indicate non‐random cargo distribution.

Comparisons between EVs and their biological sources indicate that cargo composition is shaped by both donor‐cell molecular state and selective processes operating during vesicle biogenesis and release. The extent of compositional divergence varies with donor‐cell type, physiological state, EV subtype and biogenesis pathway. Biological matrix, isolation procedure, and analytical platform can also generate apparently conflicting cargo profiles across studies. EV cargo should therefore not be assumed to be either a direct molecular replica of the parental cell or universally enriched for selected molecules. EV composition instead reflects a context‐dependent balance between parental‐cell availability and regulated cargo sorting.

## 4 Preservation of EV Protein Cargo During Storage and Handling

Most studies discussed in this section assess isolated EVs under post‐isolation storage conditions; the effects of source‐sample storage before EV isolation are considered separately in Section 4.5.

Preserving EV‐associated proteins is essential for reproducible EV‐based diagnostics, therapeutic development, and functional studies [72]. Post‐isolation handling and storage can alter EV recovery, membrane integrity, total protein content, EV‐associated protein abundance, and protein activity. These endpoints are not equivalent: preservation of total protein or canonical EV markers does not necessarily indicate maintenance of protein structure or biological function.

Physicochemical variables, including pH, repeated freeze–thaw exposure, storage temperature, storage duration, buffer composition, and protective excipients, can alter EV‐associated proteins [73, 74]. The magnitude and direction of these effects vary with EV source, isolation procedure, storage condition and analytical endpoint. This section evaluates post‐isolation preservation studies of protein cargo in EVs derived from non‐diseased cells and tissues, with attention to EV recovery, total protein abundance, EV‐associated protein levels and protein activity. Table 3 summarizes the principal findings. However, because several studies used EVs produced by immortalized cell lines, particularly HEK293T cells, these findings should be interpreted cautiously when extrapolating to primary, non‐diseased or patient‐derived EVs.

**TABLE 3:** Effects of preservation conditions on EV‐associated proteins and physical characteristics.

EV source	Isolation method	Preservation factor	Experimental conditions	Protein‐related outcome	EV physical/recovery outcome	References
HEK293T cell‐conditioned medium	UC; TFF + SEC	Storage temperature	4°C for up to 8 days	Total protein remained unchanged compared with fresh EVs	EV size was similar to fresh EVs; no significant change in EV concentration	[73]
HEK293T cell‐conditioned medium	UC; TFF + SEC	Storage temperature	4°C, −20°C or −80°C for 1 week	Total protein content was comparable across temperatures	Only minor, non‐significant differences in EV size and concentration were observed among temperatures	[73]
HEK293T cell‐conditioned medium	UC; TFF + SEC	Long‐term storage temperature	−20 or −80°C for 2–26 weeks	Total protein decreased significantly at −20°C after 8 weeks; the decrease at −80°C was not significant	EV size remained unchanged at −80°C but increased approximately 1.2‐fold at −20°C after 26 weeks; EV concentration decreased after 2 weeks at −80°C and after 3 weeks at −20°C	[73]
HEK293T cell‐conditioned medium	UC; ExtraPEG	pH	pH 4, 7 or 10 for 24 h	TSG101, Alix and Hsp70 decreased approximately twofold at pH 4 versus pH 7; Alix decreased approximately 1.6‐fold at pH 10 versus pH 7	EV concentration decreased at pH 4 and 10 compared with pH 7	[75]
HEK293T cell‐conditioned medium	UC; ExtraPEG	Freeze–thaw cycles	1–5 cycles	Marker abundance: Hsp70, TSG101 and Alix progressively decreased and reached approximately 50% of initial levels by the fifth cycle	EV concentration decreased approximately twofold after two freeze–thaw cycles compared with one cycle; no further significant decrease was reported between the second and fifth cycles	[75]
HEK293T cell‐conditioned medium	UC; ExtraPEG	Storage temperature	4°C, 37°C, 60°C, −20°C or −80°C for 24 h	Hsp70, TSG101 and Alix were approximately 2–2.3‐fold lower at −80°C, 37°C and 60°C than at 4°C	EV concentration decreased approximately 2.5‐fold at −20°C and −80°C; smaller non‐significant decreases occurred at 37°C and 60°C compared with 4°C	[75]
Human whole saliva	SEC	pH	pH 3 or 7.4 at 37°C for up to 3 h	No detectable overall protein degradation or significant loss of the assessed EV‐associated proteins; DPP IV activity decreased after exposure to pH 3	EV diameter remained unchanged at both pH values	[76]
HEK293T cell‐conditioned medium	ExoQuick	pH	pH 4, 7 or 11 for 30 min at RT	Measured total protein was approximately fivefold lower at pH 7 and tenfold lower at pH 11 than at pH 4; CD9, CD63 and Hsp70 were more abundant at pH 4 than at pH 7 and were undetectable at pH 11	NR	[77]
Human whole saliva	GFC; UC	Storage duration	4°C for 20 months	Partial protein cleavage was observed, although EV‐associated markers remained detectable throughout storage	NR	[78]
Human whole saliva	GFC; UC	Storage duration	4°C for 28 days	Total protein remained stable; DPP IV and Alix abundance was unchanged compared with fresh EVs	EV diameter remained similar to that of fresh EVs	[78]
Human whole saliva	GFC; UC	Freeze–thaw cycles	Freezing at −20°C or −80°C; repeated freeze–thaw exposure	Protein activity: a single freeze–thaw cycle at −20°C or −80°C reduced DPP IV activity by approximately 1.5‐fold compared with fresh or 4°C‐stored samples; three cycles at −20°C reduced DPP IV activity approximately sixfold	NR	[78]
Mouse bronchoalveolar lavage fluid	Differential centrifugation	Storage temperature	Fresh versus storage at 4°C or −80°C	Protein degradation and reduced CD63 abundance were observed after storage	EV size increased significantly at both 4°C and −80°C compared with fresh EVs	[79]
Cardiac progenitor cell‐derived EVs	Filtration	Storage buffer	PBS versus HBS	Higher total protein and higher Alix and Annexin A abundance were detected in HBS than in PBS	NR	[80]
Cardiac progenitor cell‐derived EVs	Filtration	Protective excipients	BSA or Tween 20	Measured total protein and EV‐associated protein marker abundance were higher in the presence of BSA or Tween 20 than in preparations without excipients	NR	[80]
Cardiac progenitor cell‐derived EVs	Filtration	Storage temperature	4°C or −80°C for 1–7 days	Total protein decreased significantly at both temperatures; EV‐associated protein marker abundance remained unchanged after 7 days	The particle‐to‐protein ratio was higher at 4°C than at −80°C	[80]
Corneal stromal stem cell‐derived EVs	TEI	Storage and lyophilization	Fresh, 4°C, −80°C, lyophilized or lyophilized with trehalose	Total protein concentration did not differ significantly among conditions; CD63, CD9 and CD81 abundance was reduced after lyophilization without trehalose compared with fresh and other tested conditions	EVs lyophilized with trehalose were smaller than EVs lyophilized without trehalose; EV concentration was lower after lyophilization than in fresh EVs and differed further with trehalose supplementation	[81]
Mouse cell‐derived EVs	SEC	Storage matrix/formulation	Serum‐containing medium versus PBS at 23°C or 37°C for 24 h, 1 week, or 2 weeks	Surface‐associated biomolecular layer: reduced loss of EV‐associated surface biomolecules in serum‐containing medium compared with PBS	Improved EV stability was reported in serum‐containing medium compared with PBS	[82]
Urine from healthy subjects	UC	Source‐sample storage before EV isolation	Fresh urine versus urine stored at 4, −20°C, or −80°C for 1 week or 7 months	Urinary EV‐associated proteins were better preserved at −80°C than at −20°C; NHE3, TSG101 and Alix were undetectable and AQP2 was reduced after storage at −20°C, whereas most assessed EV‐associated proteins were retained at −80°C	Marked EV loss occurred after storage at −20°C; approximately 86% EV recovery was reported after storage at −80°C	[83]

Abbreviations: BSA, bovine serum albumin; CM, conditioned medium; DPP IV, dipeptidyl peptidase IV; EV, extracellular vesicle; GFC, gel filtration chromatography; HBS, HEPES‐buffered saline; NHE3, sodium–hydrogen exchanger 3; NR, not reported; PBS, phosphate‐buffered saline; RT, room temperature; SEC, size‐exclusion chromatography; TEI, total exosome isolation; TFF, tangential flow filtration; UC, ultracentrifugation.

### 4.1 The Effect of Storage pH on EV Protein Cargo

Environmental pH can affect EV recovery and EV‐associated proteins, although the reported direction and magnitude of these effects vary considerably among studies. In EVs isolated from HEK293T cell‐conditioned medium, incubation at pH 4 for 24 h resulted in an approximately twofold reduction in EV concentration and in the abundance of the EV‐associated proteins TSG101, Alix and Hsp70 compared with pH 7 [75]. Exposure to alkaline pH 10 also reduced EV concentration and selected protein markers, although the effects were generally less pronounced than those observed at pH 4 [75]. These parallel changes in EV concentration and protein abundance suggest that reduced marker detection may partly reflect loss of recoverable EVs rather than direct degradation of individual proteins.

Protein activity may respond differently from protein abundance. In human salivary EVs, exposure to pH 3 for 2 h significantly reduced dipeptidyl peptidase IV (DPP IV) activity [76]. In contrast, the abundance of several assessed surface‐, membrane‐ and luminal EV‐associated proteins, including mucin 5B, immunoglobulin A (IgA), DPP IV, CD9, Alix and TSG101, showed comparatively limited changes under the tested conditions [76]. These findings illustrate that preservation of detectable protein abundance does not necessarily indicate preservation of enzymatic activity or protein function.

Contrasting results have also been reported in HEK293T‐derived EV preparations exposed to different pH conditions for 30 min. In this study, Hsp70, CD9 and CD63 were more abundant at pH 4 than at pH 7, whereas these proteins were undetectable at pH 11 [77]. Total measured protein concentration was also approximately fivefold higher at pH 4 than at pH 7 and tenfold higher than at pH 11 [77]. However, higher protein abundance under acidic conditions should not automatically be interpreted as improved preservation of protein integrity. Differences in EV recovery, aggregation, protein extractability, co‐isolated non‐vesicular proteins or altered antibody recognition may contribute to the measured protein levels.

The apparently conflicting effects of pH are likely to reflect differences in EV source, isolation method, exposure duration, temperature, and analytical endpoint. Ultracentrifugation, precipitation‐based methods, and size‐exclusion chromatography can recover EV preparations with different levels of purity and matrix‐associated components, which may influence their apparent response to pH stress. In addition, western blotting, total protein assays, and enzymatic activity measurements assess different aspects of protein preservation and should not be interpreted as equivalent outcomes. Protein localization may also influence pH sensitivity, although direct comparison of surface‐associated and intraluminal proteins remains limited.

The available evidence does not support a universally optimal pH for EV‐associated protein preservation. Future studies should separately assess EV recovery, protein abundance, structural integrity and biological or enzymatic activity to determine whether changes in protein detection reflect genuine protein preservation or secondary effects of altered EV recovery and analytical methodology.

### 4.2 The Effect of Freeze–Thaw Cycles on EV Protein Cargo

Repeated freeze–thaw cycles can adversely affect EV recovery and EV‐associated proteins. In HEK293T‐derived EV preparations, increasing the number of freeze–thaw cycles, involving freezing at −80°C for 24 h followed by thawing, was associated with progressive reductions in both EV concentration and the abundance of Alix, Hsp70 and TSG101 [75]. After two freeze–thaw cycles, EV concentration decreased to approximately 50% of that measured after the first cycle. Although no further significant reduction in EV concentration was observed between the second and fifth cycles, the abundance of EV‐associated proteins continued to decline progressively and reached approximately 50% of the initial levels by the fifth cycle [75]. These findings indicate that EV recovery and protein abundance may respond differently to repeated freeze–thaw stress.

Functional protein endpoints may be particularly sensitive to repeated freezing and thawing. In salivary EVs, a single freeze–thaw cycle at −20°C or −80°C caused only a modest reduction in dipeptidyl peptidase IV (DPP IV) activity and limited changes in the abundance of assessed EV‐associated proteins [78]. In contrast, three freeze–thaw cycles at −20°C reduced DPP IV activity by approximately sixfold. A single freeze–thaw cycle at −20°C or −80°C was associated with an approximately 1.5‐fold reduction in DPP IV activity compared with fresh or 4°C‐stored samples [78]. Thus, preservation of detectable EV‐associated proteins does not necessarily indicate preservation of enzymatic activity or EV biological function.

The mechanisms underlying freeze–thaw‐associated changes may involve membrane disruption, aggregation, fusion, adsorption to container surfaces or selective loss of EV subpopulations. These processes can reduce the number of recoverable EVs and consequently decrease the measured abundance of EV‐associated proteins without necessarily demonstrating direct degradation of all protein cargo. Repeated physical stress may additionally alter protein conformation or membrane organization and thereby impair protein activity.

The available evidence supports minimizing repeated freeze–thaw exposure during EV storage. Aliquoting EV preparations before long‐term storage may reduce repeated freezing and thawing and improve experimental reproducibility. Future studies should independently evaluate EV recovery, protein abundance, structural integrity, and biological or enzymatic activity to determine whether freeze–thaw‐associated protein loss reflects vesicle loss, protein degradation or functional impairment.

### 4.3 The Effect of Storage Temperature and Thermal Exposure on EV Protein Cargo

Storage temperature can influence EV recovery, protein abundance, and protein activity, but the optimal temperature appears to depend strongly on storage duration, EV source, and the endpoint assessed. In HEK293T cell‐derived EV preparations, short‐term storage for 24 h at 4°C was associated with higher abundance of Alix, Hsp70 and TSG101 than storage at −80°C, −20°C, 37°C or 60°C [75]. In contrast, after prolonged storage, EV‐associated proteins were better preserved at −80°C than at −20°C or 4°C [75]. These findings suggest that storage conditions suitable for short‐term handling may differ from those required for long‐term preservation.

A similar duration‐dependent pattern was reported by Görgens et al. [73]. Total protein content of HEK293T cell‐derived EVs remained largely unchanged after storage at 4°C for up to 8 days. During longer storage, protein loss became apparent at −20°C, whereas changes at −80°C were less pronounced. After 1 week, total protein levels were broadly comparable among EV preparations stored at 4°C, −20°C and −80°C and were similar to those of fresh EVs [73]. However, interpretation of total protein measurements requires caution because changes in measured protein content may reflect EV recovery, co‐isolated proteins, or differences in protein extractability and do not necessarily indicate preservation of EV protein integrity or biological function.

Source‐dependent differences have also been observed. In EVs isolated from mouse bronchoalveolar lavage fluid, storage at both 4°C and −80°C was associated with protein degradation and reduced CD63 abundance compared with fresh EV preparations [79]. The authors proposed that isolation of EVs from the surrounding lavage matrix before storage may reduce protection provided by extracellular proteins or other matrix components. However, the contribution of matrix removal to EV protein instability remains difficult to distinguish from other factors, including EV loss, membrane disruption and source‐specific differences in vesicle composition.

Cardiac progenitor cell‐derived EVs similarly showed a significant reduction in total protein content during storage at both 4°C and −80°C for 1–7 days [80]. Despite these changes, western blot analysis after 7 days revealed no obvious differences in the abundance of the assessed EV‐associated proteins. The particle‐to‐protein ratio was also higher at 4°C than at −80°C. These observations further illustrate that total protein content, EV‐associated protein abundance, and EV recovery may respond differently to storage temperature and should not be considered interchangeable measures of EV stability.

Temperature‐dependent changes in EV preservation may partly reflect alterations in membrane physical properties. Storage temperature influences lipid mobility, bilayer packing, membrane permeability and protein conformation [84]. Elevated temperatures typically enhance membrane fluidity, thereby predisposing EVs to membrane destabilization, vesicle fusion, aggregation and cargo leakage. High thermal conditions can also accelerate conformational changes in membrane‐associated and intraluminal proteins. Conversely, lower temperatures restrict lipid mobility and may reduce membrane permeability. Nevertheless, freezing introduces additional physicochemical stresses that extend beyond mechanical injury caused by ice crystals. As pure water selectively crystallizes into ice, the remaining unfrozen liquid fraction becomes progressively enriched in salts, proteins and metabolites, a phenomenon known as freeze‐concentration or cryoconcentration [85]. This localized solute accumulation markedly elevates microenvironmental ionic strength and may disrupt electrostatic interactions within the EV lipid bilayer and associated proteins [86]. Furthermore, selective crystallization of buffer components, such as dibasic sodium phosphate in phosphate‐buffered saline (PBS), can trigger substantial micro‐pH fluctuations, with local pH shifts of several units [87]. Together, hyperosmolality, elevated ionic strength, and micro‐pH shifts can promote EV surface dehydration, membrane phase separation, irreversible protein denaturation, aggregation and cargo inactivation [88]. Thus, the relationship between decreasing temperature and EV preservation is non‐linear and depends on EV lipid composition, storage matrix, freezing rate, and cryoprotective excipients.

The available evidence most consistently supports −80°C for long‐term preservation of EV preparations, whereas 4°C may be suitable for short‐term storage in selected experimental systems. These recommendations should not be generalized across all EV sources because preservation outcomes vary with storage duration, biological matrix, isolation method and analytical endpoint. Future studies should separately assess EV recovery, total protein abundance, protein structural integrity and biological activity to determine whether temperature‐associated changes reflect vesicle loss, protein degradation or functional impairment.

### 4.4 The Effect of Protective Excipients During EV Storage on Protein Cargo

Protective excipients have been investigated as strategies to reduce storage‐associated changes in EVs and their protein cargo. Lyu et al. [81] reported that EVs derived from a corneal stromal stem cell line retained total protein and EV‐associated proteins during storage at −80°C for up to 4 weeks. During lyophilization, the addition of trehalose reduced the loss of CD63, CD9 and CD81 compared with lyophilization without trehalose [81]. These findings suggest that cryoprotective excipients may improve preservation of EV‐associated proteins during drying and storage, although retention of protein markers does not necessarily establish maintenance of biological activity.

Protective effects have also been reported for bovine serum albumin (BSA) and Tween 20 in cardiac progenitor cell‐derived EV preparations. EVs stored in the presence of these excipients showed higher total protein content and greater abundance of selected EV‐associated proteins than preparations stored without excipients [80]. However, these findings should be interpreted in relation to EV recovery and formulation composition, because protein‐containing excipients such as BSA can complicate total protein‐based measurements.

The surrounding storage matrix may also influence EV preservation. Storage of mouse cell‐derived EVs in a serum‐containing medium was associated with reduced loss of the EV‐associated biomolecular corona and improved vesicle stability compared with phosphate‐buffered saline (PBS) at 23°C and 37°C [82]. Matrix‐associated proteins and other biomolecules may provide protection against proteolysis, adsorption, or membrane destabilization. Nevertheless, complex protein‐containing media can also interfere with EV characterization and cargo quantification.

Trehalose, protein‐containing formulations, and selected surfactants may improve EV preservation under specific conditions. Their effects should be evaluated using complementary measures of EV recovery, protein abundance, protein integrity, and biological function, while accounting for analytical interference introduced by the excipient itself.

### 4.5 Effect of Source‐Sample Storage on EV Protein Recovery and Cargo

Storage of the original biological sample before EV isolation represents a distinct pre‐analytical variable that can influence both EV recovery and subsequent cargo analysis. In a study of urinary EVs, freezing urine at −20°C resulted in substantial EV loss compared with freshly collected samples, whereas approximately 86% of EVs were recovered after storage at −80°C [83]. Western blot analysis detected NHE3, TSG101, Alix and aquaporin‐2 (AQP2) in EVs isolated from fresh urine. In contrast, NHE3, TSG101 and Alix were undetectable, and AQP2 abundance was reduced after storage of urine at −20°C. Storage at −80°C provided better preservation of the assessed urinary EV‐associated proteins [83].

The abundance of the EV‐associated cytosolic proteins nestin and malate dehydrogenase was also maintained after prolonged storage of urine at −80°C for up to 7 months [83]. Protease inhibitors were investigated as an additional strategy to reduce protein loss during sample handling. These findings demonstrate that storage of the source biofluid before EV isolation can substantially influence both EV recovery and the measured abundance of EV‐associated proteins.

Source‐sample storage should be distinguished from storage of isolated EVs because components of the original biological matrix may alter vesicle stability, aggregation, adsorption and protein degradation. Standardized biofluid collection and storage are essential when EV‐associated proteins are evaluated as diagnostic or prognostic biomarkers.

## 5 Preservation of EV Nucleic Acid Cargo During Storage and Handling

EV‐associated nucleic acids are of considerable interest as diagnostic biomarkers and therapeutic cargo, but their reliable analysis depends on appropriate pre‐analytical handling and storage. The EV lipid bilayer can partially protect intraluminal RNA and DNA from extracellular nucleases and other physicochemical stresses [89]. Nevertheless, disruption of EV membranes, loss of recoverable vesicles or changes in RNA extractability can substantially influence the amount and apparent integrity of nucleic acids detected after storage.

Interpretation of EV nucleic acid preservation is further complicated by differences among analytical endpoints. EV recovery, total RNA or DNA yield, transcript‐specific abundance, nucleic acid integrity and RNA‐dependent biological activity represent distinct outcomes and should not be interpreted interchangeably. Unprotected extracellular RNA can undergo rapid degradation in biological fluids, whereas EV association may increase the persistence of selected RNA species [90, 91]. However, detection of an RNA molecule after storage does not necessarily indicate preservation of its structural integrity or biological function.

This section critically evaluates the effects of storage temperature, buffer composition, freeze–thaw exposure and pH on EV‐associated nucleic acids. Particular emphasis is placed on distinguishing nucleic acid abundance and recovery from molecular integrity and functional preservation. The principal findings are summarized in Table 4. Similar to the protein‐cargo studies, a proportion of the available evidence on EV‐associated nucleic acid preservation is from immortalized cell‐line models, including HEK293T‐derived EVs. These models provide well‐controlled systems to evaluate the effects of temperature, storage formulation, pH and other physicochemical stresses on nucleic acid recovery and integrity, but EV cargo composition and storage responses may differ from EVs released by primary non‐diseased cells or physiological biofluids. Therefore, results from immortalized cell‐derived EVs should be interpreted as mechanistic and supportive rather than as direct representatives of nucleic acid preservation under physiological conditions.

**TABLE 4:** Effects of preservation and physicochemical stress on EV‐associated nucleic acids and vesicle characteristics.

EV source/material	Isolation method	Preservation or stress factor	Experimental conditions	Nucleic acid outcome	EV recovery/physical outcome	References
HEK293T/FS cell line‐derived EVs	UC; TFF + SEC	Storage temperature	4°C, −20°C, or −80°C for up to 2 years	Integrity: RNA degradation was lower at −20°C and −80°C than at 4°C. Recovery: the highest full‐length cDNA recovery was observed at 4°C	Overall EV stability was highest at −20°C; EV concentration was higher at 4°C and −80°C than at −20°C	[73]
HEK293T/FS cell line‐derived EVs	UC; TFF + SEC	Storage buffer	PBS, PBS‐A, PBS‐AT, PBS‐HAT or PBS‐HATD	RNA integrity/recovery: small‐RNA degradation was lowest in PBS‐HAT, which also yielded a higher proportion of full‐length cDNA. Longer mRNAs remained comparatively unstable. DNA recovery: no significant differences were observed among buffers	EV stability and concentration were highest in PBS‐HAT	[73]
HEK293T‐derived EVs	ExoQuick	pH	pH 4, 7, or 11 for 30 min at RT	RNA recovery: approximately fivefold higher at pH 4 than at pH 7; RNA was undetectable at pH 11	EV concentration was approximately fivefold higher at pH 4 than at pH 7	[77]
Cardiac progenitor cell‐derived EVs	Filtration	Storage buffer	HEPES versus PBS	DNA recovery: approximately 25% higher in HEPES than in PBS. RNA integrity: no significant improvement in RNA degradation was observed with HEPES compared with PBS	EV concentration was approximately 20%–30% higher in HEPES; no significant difference in EV size	[80]
Cardiac progenitor cell‐derived EVs	Filtration	Storage‐container material	Polypropylene versus glass tubes	DNA recovery: approximately 15%–20% higher in polypropylene than in glass tubes	EV concentration was approximately 15%–25% higher in polypropylene; no significant difference in EV size	[80]
Cardiac progenitor cell‐derived EVs	Filtration	Protective excipients	BSA or Tween 20	DNA recovery: approximately 10%–15% higher with protective excipients than without excipients	EV concentration was approximately 10%–20% higher with excipients; no significant difference in EV size	[80]
Plasma‐derived EVs from healthy donors	UC + SEC	Cryopreservation	−80°C for 10–12 days; PBS or DMSO‐containing cryopreservation conditions	RNA recovery/integrity: approximately 47%–52% of initial RNA was retained after cryopreservation. RIN values decreased, although electropherograms did not show equally evident RNA degradation	EV concentration was approximately 23% lower than in fresh samples; EV size did not significantly differ	[92]
Plasma‐derived EVs from healthy donors	UC + SEC	Freeze–thaw exposure	Single freeze–thaw exposure	RNA recovery: only approximately 23% of RNA was recovered relative to fresh samples; larger RNA species showed greater loss than small RNAs	NR	[92]
Serum‐derived EVs from healthy donors	UC	Short‐term storage	RT for 4 or 24 h	Abundance/stability: EV‐associated miRNA levels remained higher than those in non‐EV fractions	NR	[93]
Serum‐derived EVs from healthy donors	UC	RNase exposure	RT storage for 1, 3, or 7 h followed by RNase A treatment	Integrity/stability: miR‐16, miR‐122 and miR‐21 showed approximately 2–3‐fold greater RNase resistance in EV fractions than in non‐EV fractions	NR	[93]
Semen‐derived EVs from healthy donors	ExoQuick	Long‐term storage	−80°C for 2 versus 30 years	RNA abundance: lower RNA levels were detected after 30 years than after 2 years	No significant differences in EV size or concentration	[94]
Human urine stored before EV isolation	UC; chemical precipitation	Source‐sample preservation temperature/formulation	Preservative‐assisted RT versus −80°C for up to 6 months	miRNA abundance/recovery: selected EV‐associated miRNAs, including miR‐16, miR‐21, miR‐210 and miR‐451, remained detectable; preservative‐assisted RT storage was associated with favourable RNA and miRNA recovery compared with −80°C storage. RNA integrity was not directly assessed.	No significant differences in EV size or concentration between storage conditions; preservation of some EV‐associated protein markers was less favourable after prolonged RT storage	[95]
Human breast milk‐derived EVs	ExoQuick	Thermal exposure	100°C for 10 min	Stability: EV‐associated miRNAs showed approximately twofold greater stability than exogenous miRNAs under the same conditions	NR	[96]
Human breast milk‐derived EVs	ExoQuick	Short‐term storage temperature	26°C for 0.5–24 h	Abundance/stability: EV‐associated miRNAs remained detectable and relatively stable and were more abundant than non‐EV miRNAs throughout incubation	NR	[96]
Human breast milk‐derived EVs	ExoQuick	RNase exposure	RNase A/T1 treatment at 37°C for 1 h	Integrity/stability: EV‐associated miRNAs showed approximately 2–3‐fold greater RNase resistance than non‐EV miRNAs	NR	[96]
Human breast milk‐derived EVs	ExoQuick	Freeze–thaw cycles	Six cycles at −20°C	miRNA stability: assessed EV‐associated miRNAs remained stable after repeated freeze–thaw cycles	EV concentration decreased by approximately 50%; the reported EV size distribution shifted from 30 to 100 nm to approximately 50–70 nm	[96]
Skim bovine milk‐derived EVs	UC	Ultrasonication	185 W, 40 kHz for 90 min at 37°C followed by 60 min incubation	miRNA abundance: bta‐let‐7a‐5p, bta‐miR‐320a, bta‐miR‐200c, bta‐miR‐30d, bta‐miR‐423‐5p and bta‐miR‐148a decreased by approximately 74%–99% after ultrasonication	EV concentration was approximately 76% ± 22% lower after ultrasonication than in non‐ultrasonicated preparations	[97]
Placenta‐derived EVs from healthy pregnant women	UC + sucrose gradient	Source‐sample storage before EV isolation	Fresh plasma versus plasma stored at −80°C and thawed once	RNA abundance: no significant difference	No significant differences in EV size or concentration	[74]
Buffalo milk‐derived EVs	ExoQuick	Short‐term storage	4°C for 24 h	miRNA abundance: miR‐21 and miR‐500 decreased by approximately 10% and 15%, respectively, compared with fresh samples	EV concentration decreased by approximately 20%; EV size did not significantly differ	[98]
Buffalo milk‐derived EVs	ExoQuick	Thermal exposure	100°C for 10 min	miRNA abundance: miR‐21 and miR‐500 decreased by approximately 30% and 40%, respectively, compared with fresh samples	EV concentration decreased by approximately 60%; EV size did not significantly differ	[98]
Coffee‐derived EV‐like particles	Kit + SEC	Thermal exposure	65°C for 8 min	RNA detection: miRNAs remained detectable after heating; quantitative RNA integrity was not established	NR	[99]

Abbreviations: BSA, bovine serum albumin; DMSO, dimethyl sulfoxide; EV, extracellular vesicle; HEPES, 4‐(2‐hydroxyethyl)‐1‐piperazineethanesulfonic acid; NR, not reported; PBS, phosphate‐buffered saline; RIN, RNA integrity number; RT, room temperature; SEC, size‐exclusion chromatography; TFF, tangential flow filtration; UC, ultracentrifugation.

### 5.1 The Effect of Storage Temperature on EV Nucleic Acid Cargo

Storage temperature can influence EV‐associated nucleic acids at several distinct levels, including EV recovery, cargo abundance, cargo integrity and functional preservation. These outcomes should not be considered interchangeable. A reduction in the number of recoverable EVs does not necessarily indicate degradation of the nucleic acids contained within the remaining vesicles, whereas changes in measured miRNA abundance do not by themselves demonstrate altered RNA integrity. Similarly, preservation of RNA quantity or structural integrity does not necessarily indicate maintenance of its biological activity. Differences in the endpoints and analytical methods used across studies may therefore contribute substantially to the apparently conflicting effects of storage temperature on EV‐associated nucleic acids.

With respect to EV recovery, the influence of temperature appears to depend on EV source, storage duration, and whether isolated EVs or the original biological sample are stored. For example, cardiac progenitor cell‐derived EVs (CPC‐EVs) showed no significant differences in recovery after short‐term storage at 4°C or −80°C, suggesting that both temperatures can maintain recoverable vesicle numbers under the investigated conditions [80]. However, EV recovery should be interpreted independently from nucleic acid preservation because storage‐associated aggregation, adsorption to container surfaces, membrane disruption or selective loss of EV subpopulations may alter the number of detectable particles without proportionally affecting the nucleic acid content of the remaining EVs.

Cargo abundance represents a second, distinct endpoint. A study of urinary EV‐associated RNA reported favourable miRNA recovery after storage of pre‐cleared urine at room temperature for up to 6 months [95]. The room‐temperature samples were supplemented with a commercial urine preservative, whereas comparator samples were frozen at −80°C; EVs were isolated only after storage. The findings therefore reflect biofluid preservation before EV isolation rather than direct storage of isolated EVs. EV‐associated miR‐16, miR‐21, miR‐210 and miR‐451 remained detectable, and room‐temperature preservation was associated with favourable RNA and miRNA recovery, particularly in ultracentrifugation‐derived EV preparations [95]. These findings should not be interpreted as active miRNA upregulation or unequivocal preservation of EV structural integrity. Long‐term room‐temperature storage showed less favourable preservation of some EV‐associated protein markers, indicating that RNA recovery and EV structural preservation may respond differently to storage conditions. Differences in measured miRNA abundance may additionally reflect storage‐ or isolation‐dependent recovery of distinct EV populations. This study demonstrates that preservative‐assisted room‐temperature storage of urine can support downstream EV RNA analysis, but its findings should not be generalized to isolated EV storage or room‐temperature storage of untreated urine.

Assessment of RNA integrity provides information that is conceptually different from RNA abundance. Studies have reported that storage at 4°C may result in higher full‐length cDNA ratios, whereas broader measures of EV stability may favour −20°C or −80°C [73]. Such findings demonstrate that a storage condition associated with greater transcript detectability does not necessarily provide superior preservation of EV structure or total RNA integrity. Interpretation is further complicated by the analytical approach used to evaluate RNA quality. The RNA integrity number (RIN), which was developed primarily for cellular RNA containing abundant ribosomal RNA, may have limited applicability to EV‐associated RNA because EV preparations often contain predominantly small RNA species and relatively low levels of intact ribosomal RNA. Consequently, RIN values may not accurately reflect EV RNA degradation and should be interpreted together with electrophoretic profiles, RNA fragment‐size distributions and transcript‐specific measurements.

Evidence regarding functional preservation of EV‐associated nucleic acids after storage remains comparatively limited. Detection of an abundant or apparently intact miRNA does not demonstrate that the RNA remains biologically active, is efficiently delivered to recipient cells, or retains its capacity to regulate target genes. Direct functional assessment, such as reporter assays, target‐gene expression analysis, or evaluation of RNA‐dependent phenotypic responses in recipient cells, is required to establish functional preservation. Accordingly, storage conditions considered suitable on the basis of EV recovery or RNA abundance should not automatically be assumed to preserve the biological activity of EV nucleic acid cargo.

Divergent temperature‐dependent findings across studies likely arise from differences in EV source, biological matrix, storage duration, isolation method and analytical endpoint. Source‐specific membrane lipid and protein composition may also influence EV susceptibility to thermal stress. Freezing, particularly at −80°C, remains the most consistently supported strategy for long‐term preservation, whereas 4°C may be suitable for short‐term storage of selected EV preparations. Preservative‐assisted room‐temperature storage has supported downstream EV‐associated RNA analysis in selected urine‐based applications, but these observations should not be generalized to untreated biofluids, isolated EVs or other RNA endpoints. Future studies should independently assess EV recovery, nucleic acid abundance, RNA integrity and biological function to define source‐ and application‐specific storage conditions.

### 5.2 The Effect of Storage Buffer and Formulation on EV Nucleic Acid Cargo

Storage‐medium composition can influence EV‐associated nucleic acids at the levels of EV recovery, nucleic acid abundance, and cargo integrity. These outcomes must be distinguished when storage formulations are compared: a buffer that improves particle recovery does not necessarily preserve RNA integrity, and increased DNA or RNA recovery may reflect improved extraction or EV retention rather than molecular stabilization. Formulation excipients such as albumin, trehalose and dimethyl sulfoxide (DMSO) may further modify storage‐associated effects.

The influence of storage formulation on RNA integrity was examined in HEK293T cell‐derived EVs isolated by ultrafiltration and size‐exclusion chromatography (SEC) [73]. Among the tested formulations, phosphate‐buffered saline (PBS) alone was associated with greater degradation of small RNAs at 4°C, −20°C and −80°C. Supplementation of PBS with human serum albumin and trehalose (PBS‐HAT), or with these components together with DMSO (PBS‐HATD), improved small RNA preservation [73]. PBS‐HAT also yielded a higher proportion of full‐length complementary DNA (cDNA), supporting improved preservation of selected RNA species. However, longer messenger RNAs (mRNAs) remained comparatively unstable under the investigated storage conditions. These findings indicate that improved preservation of small RNA does not necessarily extend equally to all RNA classes and that cargo‐specific endpoints should be considered when evaluating storage formulations.

In contrast, studies of cardiac progenitor cell‐derived EVs (CPC‐EVs) illustrate the distinction between EV recovery, nucleic acid abundance, and RNA integrity. Replacing PBS with HEPES buffer was associated with an approximately 20%–30% increase in recoverable EV concentration and an approximately 25% increase in DNA recovery [80]. However, HEPES did not significantly improve RNA degradation relative to PBS [80]. Thus, the higher particle and DNA recovery observed with HEPES should not be interpreted as evidence of superior RNA preservation. Rather, these findings suggest that the effect of the storage medium may differ according to the measured cargo and analytical endpoint.

These findings are not necessarily contradictory. The studies used different EV sources and isolation procedures and, crucially, assessed different preservation outcomes. The HEK293T EV study primarily evaluated RNA size distribution and cDNA recovery, whereas the CPC‐EV study assessed EV recovery and DNA and RNA yield [73, 80]. EV source, matrix composition and formulation may additionally shape storage responses. Direct comparisons between PBS‐HAT and HEPES therefore cannot establish the universal superiority of either formulation.

The contribution of individual formulation components should also be interpreted cautiously. Albumin and trehalose may improve preservation through combined effects on EV colloidal and membrane stability, whereas DMSO may reduce freezing‐associated damage. However, improved RNA recovery in the presence of these excipients does not by itself demonstrate preservation of RNA biological activity. Direct functional assessment, such as delivery of EV‐associated RNA to recipient cells and subsequent target‐gene regulation, remains necessary to establish functional preservation.

The available evidence indicates that storage buffer and formulation should be selected according to EV source, nucleic acid species of interest, storage temperature, storage duration and downstream analytical application. PBS alone may be suboptimal for preservation of small EV‐associated RNAs under some conditions, but current evidence does not support its universal exclusion as an EV storage or dilution buffer. Formulations containing stabilizing excipients, such as albumin and trehalose, may improve small RNA preservation, whereas HEPES may enhance EV and DNA recovery in specific preparations. Future studies should separately assess EV recovery, nucleic acid abundance, nucleic acid integrity and RNA‐dependent biological activity to define formulation‐specific preservation strategies.

### 5.3 The Effect of Freeze–Thaw Cycles on EV Nucleic Acid Cargo

Freeze–thaw cycles can affect EV‐associated nucleic acids by altering EV recovery, RNA abundance, and RNA integrity, although the magnitude of these effects varies substantially among studies. In plasma‐derived EV preparations, a single freeze–thaw cycle was associated with a marked reduction in total RNA yield, with only approximately 23% recovered relative to fresh samples [92]. By contrast, uninterrupted cryopreservation at −80°C for up to 12 days retained approximately 47%–52% of the initial RNA level [92]. These observations indicate that repeated physical disruption during freezing and thawing may be more detrimental to RNA recovery than uninterrupted frozen storage.

The reduction in RNA yield after freeze–thaw exposure should not, however, be considered synonymous with RNA degradation. Loss of recoverable EVs, membrane disruption, aggregation or selective loss of EV subpopulations may reduce the amount of RNA extracted without necessarily indicating structural degradation of all remaining RNA molecules. The RNA extraction method may further influence the measured outcome. Comparison of RNeasy and miRNeasy protocols suggested differential recovery of RNA species, with larger RNAs showing greater loss than small RNAs [92]. Thus, changes in total RNA yield may reflect both storage‐associated effects and methodological differences in RNA recovery.

Assessment of RNA integrity also produced apparently divergent findings. Although RNA integrity number (RIN) values decreased significantly in cryopreserved plasma samples, corresponding electropherograms did not show equally evident RNA degradation [92]. This discrepancy highlights the limitations of applying RIN alone to EV‐associated RNA, particularly because EV preparations commonly contain relatively low levels of ribosomal RNA and are enriched in shorter RNA species. Electrophoretic profiles, RNA size distributions and transcript‐specific measurements should therefore be considered together when assessing the effects of freeze–thaw cycles on EV RNA integrity.

In contrast to the marked RNA loss reported for plasma‐derived EV preparations, EV‐associated miRNAs in human breast milk showed comparatively high resilience to repeated freeze–thaw exposure and other physicochemical stresses [96]. Specific miRNAs, including miR‐148a‐3p, miR‐30b‐5p and miR‐200a‐3p, remained readily detectable after RNase treatment, heating at 100°C for 10 min, and repeated freeze–thaw cycles [96]. These findings suggest that freeze–thaw susceptibility is dependent on the biological source and the RNA species examined. However, higher or preserved miRNA abundance should not automatically be interpreted as maintenance of the overall EV RNA cargo or EV structural integrity. Differences in EV isolation procedures, surrounding biological matrices and analytical endpoints may contribute to the contrasting findings between plasma‐ and milk‐derived EV preparations.

Cryoprotective strategies may reduce freeze–thaw‐associated RNA loss. The addition of 10% dimethyl sulfoxide (DMSO), together with controlled freezing and thawing rates, improved RNA recovery in cryopreserved samples [92]. Nevertheless, the suitability of DMSO for prolonged storage at −80°C has been questioned because its thermal properties may not provide optimal vitrification under these conditions [100]. Therefore, the benefits of cryoprotectants should be evaluated according to storage temperature, duration, EV source and intended downstream application rather than assumed to provide universal protection.

Repeated freeze–thaw exposure should be minimized because it can reduce EV recovery and RNA yield. The extent of nucleic acid damage remains source‐ and endpoint‐dependent, and changes in RNA abundance should be distinguished from loss of RNA integrity. Aliquoting EV samples before long‐term storage may reduce repeated freeze–thaw exposure. Future studies should assess EV recovery, RNA abundance, RNA integrity and RNA‐dependent biological activity as separate outcomes to determine whether preserved RNA remains functionally competent after cryopreservation.

### 5.4 The Effect of Storage pH on EV Nucleic Acid Cargo

Environmental pH may influence EV recovery and the measured abundance of EV‐associated nucleic acids. In HEK293T‐derived EV preparations, exposure to pH 4 for 30 min was associated with approximately fivefold higher RNA recovery than exposure to pH 7, whereas RNA was undetectable at pH 11 [77]. EV concentration showed a similar pattern, with approximately fivefold higher particle recovery at pH 4 than at pH 7. These parallel changes suggest that differences in measured RNA yield may, at least partly, reflect altered EV recovery rather than direct stabilization or degradation of the RNA cargo.

Accordingly, higher RNA abundance under acidic conditions should not automatically be interpreted as improved nucleic acid integrity. Changes in membrane stability, EV aggregation, particle recovery or RNA extractability may influence the amount of RNA detected. In contrast, the absence of detectable RNA at highly alkaline pH may result from EV disruption, RNA degradation, reduced EV recovery or a combination of these effects. Because RNA integrity and biological activity were not directly assessed, the available findings primarily provide evidence regarding RNA recovery rather than functional preservation of EV‐associated nucleic acids.

The effects of pH are also likely to depend on EV source, exposure duration, surrounding matrix, isolation procedure and analytical endpoint. Current evidence is therefore insufficient to define a universally optimal pH for EV nucleic acid preservation. Future studies should assess EV recovery, RNA abundance, RNA integrity and RNA‐dependent biological activity independently to distinguish improved cargo recovery from genuine preservation of nucleic acid integrity and function.

## 6 Donor‐Cell Conditions as Distinct Determinants of EV Cargo

Pre‐isolation biological and culture conditions can alter EV cargo composition and membrane properties. Donor‐cell state at the time of EV secretion, including oxygenation, replicative history and exposure to bioactive stimuli, dictates the baseline EV cargo profile and should therefore be treated as an essential confounding variable when interpreting post‐isolation stability assays. These donor‐cell conditions should be distinguished from post‐isolation storage stress when changes in EV protein, nucleic acid, or lipid cargo are interpreted.

### 6.1 Effect of Hypoxic Preconditioning on EV Protein Cargo

Hypoxia can alter the protein composition of EVs by modifying the physiological state of donor cells. Lu et al. [101] reported no significant differences in the expression of the EV‐associated proteins CD63, TSG101 and CD81 between EVs derived from normoxia‐conditioned human mesenchymal stromal cells (norm‐EVs) and those derived from hypoxia‐conditioned cells (hypo‐EVs). In contrast, Paw et al. [102] exposed human induced pluripotent stem cells (hiPSCs) to normoxia (21% O_2_) or hypoxia (5% or 3% O_2_). EVs derived under both hypoxic conditions showed increased total protein levels compared with norm‐EVs, with approximately 1.6‐ and 1.4‐fold increases in EVs generated at 5% and 3% O_2_, respectively. Increased CD81 expression was also observed in hypoxia‐derived EVs [102]. Similarly, EVs derived from hypoxia‐conditioned mouse bone marrow mesenchymal stromal cells showed higher levels of TSG101 and Alix than those released under normoxic conditions [103].

The divergent effects of hypoxia on EV‐associated proteins may reflect differences in donor‐cell type, oxygen concentration, duration of hypoxic exposure and analytical endpoint. Changes in EV marker abundance after hypoxic preconditioning should not be interpreted as evidence of improved EV preservation. They more likely reflect hypoxia‐dependent alterations in EV biogenesis, secretion or cargo sorting.

### 6.2 Effect of Hypoxic Preconditioning on EV Nucleic Acid Cargo

Hypoxic preconditioning can also modify the nucleic acid cargo of EVs, particularly miRNAs involved in adaptive and regenerative signalling. In one study, human umbilical cord‐derived mesenchymal stromal cells (huMSCs) exposed to 1% O_2_ for 48 h released EVs containing approximately threefold higher levels of miR‐214 than EVs derived from normoxic cells [104]. Because miR‐214 is involved in angiogenic regulation, this alteration in EV cargo was associated with enhanced biological activity.

Consistent with this observation, treatment of human umbilical vein endothelial cells (HUVECs) with EVs derived from hypoxia‐preconditioned huMSCs (MSChyp‐EVs) significantly enhanced endothelial migration and tube formation compared with untreated cells or cells treated with EVs derived from normoxic MSCs. Increased tube length and branching further supported the enhanced pro‐angiogenic activity of MSChyp‐EVs [104].

These findings separate hypoxic preconditioning from post‐isolation storage stress. Adverse storage conditions may compromise EV integrity or promote cargo loss, whereas hypoxia acts on viable donor cells before EV release and can activate adaptive transcriptional and cargo‐sorting pathways. Increased EV‐associated miRNA abundance after hypoxic preconditioning should therefore be interpreted as altered cargo loading rather than improved nucleic acid stability. The magnitude and functional consequences of this response are likely to depend on donor‐cell source, hypoxia severity and duration, and the biological and analytical endpoints examined.

### 6.3 Effect of Donor‐Cell Passage on EV Nucleic Acid Cargo

Donor‐cell replicative state can also influence EV cargo independently of post‐isolation storage. In bone marrow‐ and adipose‐derived mesenchymal stromal cells, increasing passage number was associated with changes in EV‐associated miRNA profiles, including increased miR‐145 and miR‐100 and decreased miR‐214, miR‐21 and miR‐126 in later‐passage EV preparations [105]. EV size and concentration also changed with increasing passage number. These findings indicate that donor‐cell culture history can alter baseline EV composition and should be controlled when evaluating storage‐associated changes in EV nucleic acid cargo.

### 6.4 Effect of Bioactive Donor‐Cell Conditioning on EV Dynamics

Donor‐cell exposure to bioactive molecules can modify EV release and cargo independently of post‐isolation storage. In bovine cumulus cells, melatonin treatment changed exosome dynamics by reducing exosome size, altering the expression of exosome‐related markers including CD63 and Rab27b, and increasing unsaturated fatty‐acid proportions, including arachidonic acid, oleic acid and linoleic acid [106]. These findings support the broader concept that donor‐cell metabolic and signalling conditions can influence EV biogenesis and composition before vesicle isolation.

## 7 Conclusion and Future Perspective

Extracellular vesicles from non‐diseased cells and tissues are promising platforms for diagnostic, drug‐delivery, immunomodulatory, and regenerative applications. Their experimental and clinical use requires preservation of vesicle number, morphology, molecular cargo integrity and biological activity. Post‐isolation conditions, including temperature, pH, repeated freeze–thaw cycles, storage buffer and protective excipients, differentially affect EV‐associated proteins and nucleic acids.

A major limitation of the current literature is the frequent use of EV recovery, cargo abundance, cargo integrity and functional preservation as interchangeable measures of EV stability. Increased protein or RNA recovery does not necessarily indicate preservation of molecular integrity, and detection of apparently intact cargo does not establish maintenance of biological activity. These endpoints should therefore be assessed independently and integrated when defining optimal storage conditions.

For practical application, storage conditions should be selected according to the intended downstream endpoint and storage duration. When short‐term preservation is required, 4°C may be suitable for selected EV preparations, particularly when protein abundance is the primary endpoint. For long‐term preservation of protein or RNA cargo, −80°C currently has the most consistent support. Repeated freeze–thaw cycles should generally be minimized, and aliquoting before long‐term storage is advisable. If RNA integrity or transcript‐specific analysis is the main endpoint, buffer composition should be considered carefully, because PBS alone may be suboptimal in some systems, whereas stabilizing formulations may improve RNA preservation. For functional assays, fresh EVs should be used whenever feasible; when storage is unavoidable, aliquoted −80°C‐stored preparations are generally preferable to repeated freeze–thaw exposure. The effects of −20°C storage, pH and buffer composition remain more source‐ and endpoint‐dependent, while protective excipients such as trehalose or protein‐containing formulations may improve preservation under specific conditions. A simplified endpoint‐guided workflow for selecting EV storage conditions is provided in Figure 1.

**FIGURE 1:**
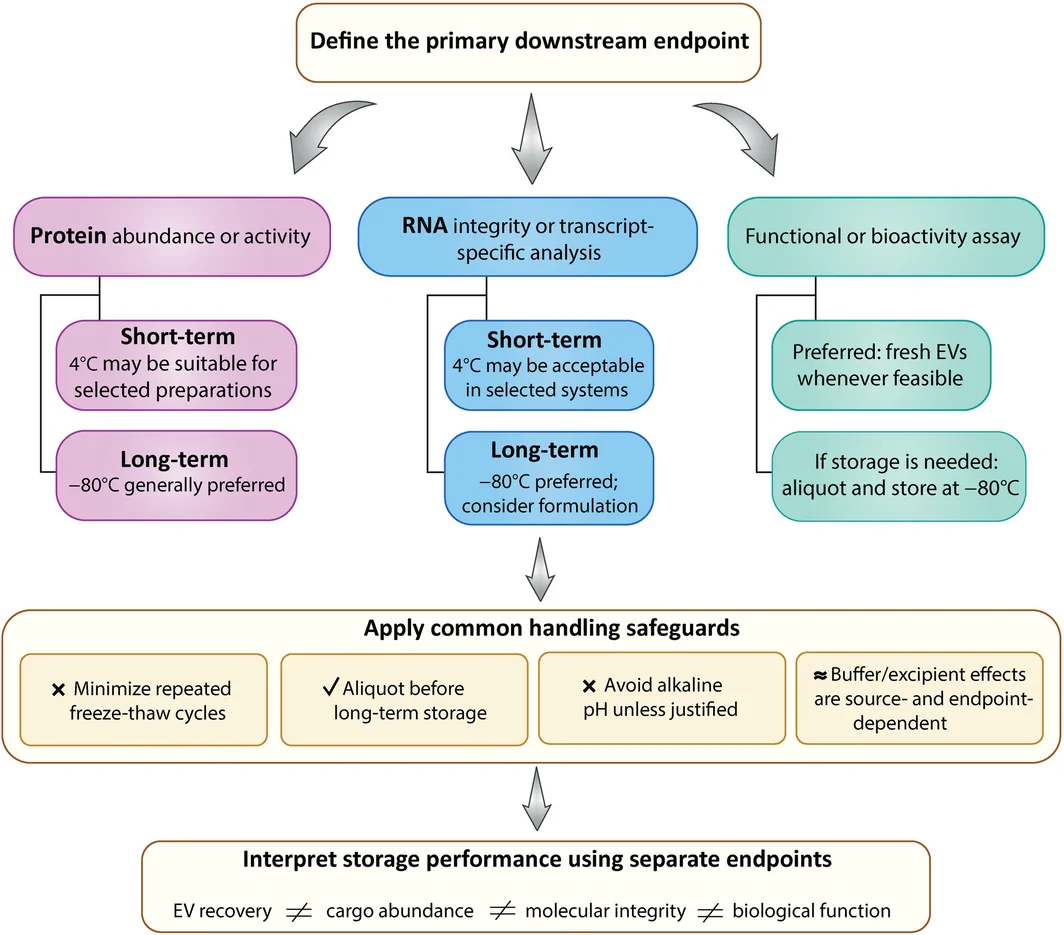
Endpoint‐guided workflow for extracellular vesicle storage and handling. General recommendations should be adapted to EV source, isolation method, storage duration, and analytical endpoint.

Donor‐cell conditions, including hypoxic preconditioning, replicative passage and exposure to bioactive molecules, can alter EV biogenesis and cargo loading before vesicle release and should be distinguished from post‐isolation preservation stress.

Future studies should apply standardized EV isolation and characterization procedures and clearly distinguish storage of the original biological matrix from storage of isolated EVs. Parallel assessment of EV recovery, cargo abundance, molecular integrity and biological function will be essential for source‐ and application‐specific preservation protocols. Such standardization will strengthen the reproducibility and translational development of EV‐based diagnostics and therapeutics.

## Author Contributions

**Khadijeh Abbasi:** writing – original draft, visualization. **Mina Mohammadinasr:** writing – original draft. **Roza Motavalli:** writing – original draft. **Narges Arianfar:** writing – original draft. **Attabak Toofani‐Milani:** writing – original draft. **Moein Shirzad:** writing – original draft. **Vahid Hosseini:** conceptualization, supervision, writing – review and editing, visualization, writing – original draft. **Masoud Darabi:** conceptualization, supervision, writing – review and editing, visualization, writing – original draft. All authors read and approved the final manuscript.

## Funding

This work was supported in part by the Brigitte and Dr. Konstanze Wegener Foundation [grant number 126]. Masoud Darabi received support from the Annelise Asmussen Foundation.

## Ethics Statement

The authors have nothing to report.

## Consent

The authors have nothing to report.

## Conflicts of Interest

The authors declare no conflicts of interest.

## Data Availability

Data sharing not applicable to this article as no datasets were generated or analysed during the current study.
